# Quantification and Distribution of Omega-3 Fatty Acids in South Pacific Fish and Shellfish Species

**DOI:** 10.3390/foods9020233

**Published:** 2020-02-21

**Authors:** Miguel Ángel Rincón-Cervera, Valeria González-Barriga, Jaime Romero, Rodrigo Rojas, Sandra López-Arana

**Affiliations:** 1 Instituto de Nutrición y Tecnología de los Alimentos (INTA), Universidad de Chile, Avda. El Líbano 5524, Macul, Santiago 7830490, Chile; valeria.gonzalez@inta.uchile.cl (V.G.-B.); jromero@inta.uchile.cl (J.R.); 2 Departamento de Acuicultura, Facultad de Ciencias del Mar, Universidad Católica del Norte, Larrondo 1281, Coquimbo 1781421, Chile; rrojas@ucn.cl; 3 Departamento de Nutrición, Facultad de Medicina, Universidad de Chile, Avda. Independencia 1027, Independencia, Santiago 8380453, Chile; sandralopez@med.uchile.cl

**Keywords:** Omega-3 fatty acids, eicosapentaenoic acid, docosahexaenoic acid, fish, shellfish, phospholipids

## Abstract

Fatty acid composition and distribution in edible species of fish and shellfish captured in the South Pacific were studied, with a focus on n-3 long-chain polyunsaturated fatty acids (n-3 LCPUFA). Fatty acids were quantified using gas-chromatography coupled with flame ionization detection (GC-FID), and the distribution of different fatty acids within lipid classes (neutral and polar lipids) was achieved after oil extraction using the Folch method and separation of lipid classes via solid-phase extraction for further GC-FID analysis. Red cusk-eel was the fish species with the lowest content of both EPA and DHA (40.8 and 74.4 mg/100 g, respectively) whereas mackerel contained the highest amount (414.7 and 956.0 mg/100 g for EPA and DHA, respectively). Sea squirt was the shellfish species with the highest content of EPA and DHA (375.0 and 165.7 mg/100 g, respectively) whereas the lowest amount of EPA + DHA was found in Chilean abalone (63.6 mg/100 g). PUFA were mostly found in neutral or polar lipids depending on the studied species. Indexes used to discuss the nutritional quality of lipids (PUFA/SFA, n-6/n-3 ratio and the hypocholesterolemic/hypercholesterolemic fatty acid index) were calculated and reported in the manuscript. This information provides a novel nutritional insight which may be useful to help nutritionists and other health professionals give more accurate counseling for the population to reach the recommended daily intakes of EPA and DHA.

## 1. Introduction

Omega-3 (or n-3) polyunsaturated fatty acids (n-3 PUFA) are key nutrients widely recognized for their relevant healthy effects. Eicosapentaenoic acid (EPA, 20:5n-3) and docosahexaenoic acid (DHA, 22:6n-3) are the most known n-3 PUFA because of their essential role in many physiological mechanisms in the human organism. They are essential for proper fetal development, and their consumption is associated with improved cardiovascular health [1]. DHA is mainly found in the brain and the retina, and it plays a key role for the development and maintenance of the visual and cognitive systems [2]. Dietary intake of n-3 PUFA, especially EPA and DHA, is associated with a lower risk of depression [3]. Furthermore, both n-3 PUFA are metabolic precursors of lipid mediators with potent anti-inflammatory effects such as E-series resolvins (RvE1 and RvE2) derived from EPA and D-series resolvins (RvD1 and RvD2), D1 protectin (PD1) and maresins derived from DHA [4].

Although EPA and DHA can be synthesized by the human organism from their precursor alpha-linolenic acid (ALA, 18:3n-3), the conversion rate is very low because of the limited activity of the enzyme Δ6-desaturase, which is involved in the metabolic pathway [5]. Thus, both n-3 PUFA must be preferentially provided with the diet, marine foods (fish and seafood) being the most important sources [6,7,8].

Recommended daily intake of EPA + DHA are set between 100 and 250 mg for children up to 10 years, 250 mg for healthy adults, and 300 mg for pregnant women (of which at least 200 mg must be DHA), according to the Food and Agriculture Organization (FAO) [9]. However, in Latin America countries, and particularly in Chile, little is known regarding the content of n-3 PUFA in marine products which are usually consumed by the population. Our group has recently reported the fatty acid composition and distribution in seven fish species captured in Northern Chile (Coquimbo region): Chilean hake (*Merluccius gayi gayi*), Pacific pomfret (*Brama australis*), Peruvian morwong (*Cheilodactylus variegatus*), Pacific sandperch (*Prolatilus jugularis*), Chilean jack mackerel (*Trachurus murphyi*), chub mackerel (*Scomber japonicus*) and fine flounder (*Paralycthis adpersus*) [7]. This work revealed that all studied fish species contained more than 200 mg EPA + DHA/100 g raw fillet, and that Peruvian morwong, Chilean jack mackerel and Pacific sandperch were particularly rich sources, ranging from 314 to 440 mg EPA + DHA/100 g raw fillet. We reported also that DHA was mainly found in the phospholipid (PL) fraction of fish lipids in all cases, which is interesting from a nutritional point of view, as some studies have pointed to the higher bioavailability of PUFA when they are supplied as PL instead of as triacylglycerols (TAG) [10,11].

However, as previously mentioned, there is a lack of information regarding fatty acid composition of other marine foods such as shellfishes, which are regularly consumed by the Chilean population. The current work tries to fill the gap by providing novel data regarding fatty acid composition and distribution of several shellfish species but also of some fish species which were not included previously, in an attempt to further contribute to improve the nutritional characterization of marine foods consumed by the Chilean population. Additionally, several indexes used to assess the nutritional quality of marine lipids have been calculated and discussed. This is a cross-sectional and prospective study because factors such as water temperature, geographical location, capture season and feed compositions, which are known to be able to modify lipid composition of fishes [12,13], were not considered.

## 2. Materials and Methods

### 2.1. Solvents and Reagents

Unless otherwise stated, all solvents and reagents used in this work are from Merck (Darmstadt, Germany).

### 2.2. Samples

Eight different fish species (*n* = 4 for each species) and nine shellfish species (*n* = 10 for each species) were provided by local fishermen at Coquimbo’s fishing port (Coquimbo Region, Chile) (Table 1). Fishes were captured in March 2018, except jack mackerel, Chilean hake, and mackerel, which were captured in June 2018, whereas shellfishes were captured between May and July 2018. Edible parts of fishes (fillets) and shellfishes (meat, and gonads in the case of sea urchin) were sampled after collection and placed in polyethylene bags. Bags were vacuum-sealed, and samples were frozen and delivered to the laboratory facilities where they were kept at −80 °C until further processing.

### 2.3. Determination of Total Fat and Fatty Acid Quantification

Samples were thawed in water at room temperature and then homogenized in a domestic food processor. Composites were obtained, and aliquots were collected for lipid extraction in triplicate as described in Reference [7]. Fatty acid composition of extracted lipids was obtained after derivatization to fatty acid methyl esters (FAME) as described in Reference [7]. FAME were identified after analysis using gas-chromatography coupled with flame ionization detector (GC-FID) according to their respective retention times compared with two known analytical standards (37 component FAME Mix and PUFA mix 3 from Supelco, Sigma-Aldrich). Fatty acid quantification was carried out using the following equation: C_FA_ = (m_IS_ × A_FA_ × RRF_FA_)/(1.04 × m_fish_ × A_IS_), where C_FA_ is the concentration of the fatty acid in mg/g, m_IS_ is the weight of the internal standard, A_FA_ is the fatty acid peak area in the GC spectrum, RRF_FA_ is the relative retention factor for each fatty acid [14], 1.04 is the correlation factor between fatty acids and fatty acid methyl esters, m_fish_ is the weight of the fish tissue, and A_IS_ is the internal standard peak area in the GC spectrum.

### 2.4. Nutritional Quality Indexes of Lipids

Three indexes were calculated for each fish and shellfish species based on fatty acid composition. PUFA/SFA refers to the ratio between polyunsaturated and saturated fatty acids, n-6/n-3 was calculated as Σ n-6 PUFA/Σ n-3 PUFA, and the index of hypocholesterolemic/hypercholesterolemic fatty acids (HH) was calculated as follows [15]:

HH = (18:1n-9 + 18:2n-6 + 18:3n-3 + 20:4n-6 + 20:5n-3 + 22:5n-3 + 22:6n-3)/(14:0 + 16:0)

### 2.5. Fatty Acid Distribution among Lipid Classes

Total lipids were extracted from fish fillets and shellfish meat using the same method explained above using 5 g composite but without adding internal standard. Lipids were then fractionated into neutral lipids (NL), glycolipids (GL) and PL using solid-phase extraction (SPE) cartridges as described in [7]. Resulting lipid fractions were weighed after solvent removal in a rotary evaporator under vacuum. All fractions were derivatized to FAME and then fatty acid profiles were obtained by GC-FID. Methyl tricosanoate was used as an internal standard for quantification.

### 2.6. Statistical Analysis

Data regarding fatty acid composition were reported as mean ± standard deviation for three replicates of each sample. Lipid fractionation and fatty acid distribution among lipid classes were reported as percentages and 95% confidence intervals. Data were analyzed using tests of proportions to determine in which lipid classes DHA and EPA were more concentrated. The statistical analysis was performed using Stata version 14.

## 3. Results and Discussion

### 3.1. Lipid Amount and Fatty Acid Content in Fish

Lipid amount and fatty acid content in fish fillets are shown in Table 2. Lipid content ranged between 0.69 and 6.44 g/100 g raw fillet in red cusk-eel and mackerel, respectively. Red cusk-eel, palm ruff, corvina drum and Chilean hake contained less than 2 g lipids/100 g raw fillet, and therefore they are classified as lean fishes [16]. Chilean sandperch and jack mackerel are classified as low-fat fishes (2–4 g lipids/100 g raw fillet), whereas yellowtail amberjack and mackerel are considered fatty fishes as they contained more than 4 g lipids/100 g raw fillet.

In our previous work, Chilean hake, jack mackerel, and mackerel were also analyzed. Comparing the lipid content in these species in our current and former work, it was found that Chilean hake showed similar values (1.29 vs. 1.42 g/100 g raw fillet), but jack mackerel and mackerel showed significantly higher lipid amounts in the current work (3.77 vs. 1.59 and 6.44 vs. 1.25 g/100 g raw fillet respectively). It is known that lipid amount in fishes can vary substantially depending on the catch season, among other factors, which is in turn affected by water temperature or fish feeding type. In our previous study, fishes were captured in March, at the end of the summer season, whereas in the current work, mackerel and jack mackerel were captured in June, at the beginning of winter season, when water is colder and fishes usually increase their lipid amount. This could explain why the lipid amount was higher in these cases. Furthermore, the spawning season has probably also a significant influence on the difference in lipid content found in mackerel and jack mackerel. In the current work, both species were captured in winter, that is, out of their spawning season which takes place in spring and summer [17,18]. However, in our previous study, they were captured in summer. Lower lipid amounts during the spawning season have been described for many fish species because of fasting, the ripening of reproductive tissues and mobilization of lipid stores from muscle and liver tissues to the gonads [19]. There is existing evidence supporting this fact; a study carried out with Mediterranean fishes reported that jack mackerel can quadruple its total lipid content from one season to another, whereas mackerel is able to increase eightfold its lipid amount between spring and winter [20].

The highest and lowest amounts of both EPA and DHA were found in mackerel and red cusk-eel respectively, which are the oiliest and leanest fishes, respectively, among all fishes studied in this work (Table 2). Regarding mackerel and jack mackerel, which were also studied in our previous study [7], the amounts of EPA + DHA are now higher (5.0 times higher in mackerel and 2.3 times higher in jack mackerel), but it does not mean that their oils are richer in these n-3 PUFA, but that the oil content is higher (5.2 times for mackerel and 2.4 times for jack mackerel), probably because of the difference in the capture season, as previously explained. This way, the ratio (EPA + DHA)/lipid content is unchanged in both fish species.

DHA was found in higher amounts than EPA in most of the analyzed fish species, with a DHA/EPA ratio ranging from 4.93 in yellowtail amberjack (761.35 and 154.41 mg/100 g raw fillet for DHA and EPA, respectively) to 0.78 in Chilean sandperch (221.73 and 285.87 mg/100 g raw fillet for DHA and EPA), which was the only fish species with a higher amount of EPA than DHA (Table 2). According to international recommendations, daily intake of EPA + DHA should reach at least 250 mg for healthy adults. Based on our results, almost all the studied species in this work except red cusk-eel provide >250 mg of EPA + DHA per serving size (100 g). The minimum recommended amount of EPA + DHA provided by the diet for healthy adults is currently set at 1750 mg per week, and that way, 1.3 servings of raw mackerel per week (127.4 g) can supply that amount of EPA + DHA (18.2 g flesh/day) (Table 2), followed by yellowtail amberjack (1.9 servings/week, or 191.1 g flesh). In these two cases, two servings per week would be enough to provide the recommended minimal amounts of both n-3 PUFA. The fact that DHA is more abundant than EPA in most analyzed fishes is relevant because this n-3 PUFA is mainly found in the brain, being essential for the proper development and maintenance of this organ. DHA modulates neuroinflammation, cell survival, neuronal homeostasis and neurotransmission [21]. This way, fish consumption, especially those fishes which provide higher amounts of DHA, must be encouraged mainly in several sensitive population groups with special requirements of DHA, such as children, pregnant women and the elderly. Diets in most Western countries are rich in n-6 PUFA, providing a 20:1 n-6/n-3 PUFA ratio approximately. A healthy n-6/n-3 PUFA ratio is considered to be 5:1, and therefore there is an urgent need to consume foods rich in n-3 PUFA to equilibrate this balance. Amounts of n-3 PUFA were much higher than those of n-6 PUFA in analyzed fish fillets, thus providing very low n-6/n-3 PUFA ratios (between 0.08 for yellowtail amberjack and 0.22 for palm ruff), which is highly desirable from a nutritional perspective (Table 2). This fact was also found in our previous work, and it is a common finding in many other marine organisms because of the abundance of n-3 LCPUFA (mainly EPA and DHA) in comparison with n-6 PUFA in the marine food web [13,22]. Actually, fishes analyzed in this work showed lower n-6/n-3 PUFA ratios than some fish species in Sri Lanka and Japan [23] and similar values than fishes in Brazil [24] and China [15]. This way, marine fish consumption is one of the most effective ways to achieve a more balanced n-6/n-3 PUFA ratio in the human organism.

Regarding saturated (SFA) and monounsaturated fatty acids (MUFA), palmitic acid (16:0) and oleic acid (18:1n-9) were the main fatty acids within SFA and MUFA respectively in all fish species, which is in agreement with previous reports [7,25,26]. But more relevant from a nutritional point of view is the PUFA/SFA ratio, which is considered a marker for cardiovascular health. SFA are associated with an increase of total cholesterol and LDL-cholesterol in serum [27], and a low PUFA/SFA ratio is linked to a higher prevalence of cardiovascular disease. PUFA/SFA ratio in analyzed fish species ranged between 0.80 (Chilean sandperch) and 1.60 (red cusk-eel) (Table 2). PUFA/SFA values higher than 0.40 are considered desirable from a nutritional point of view to help prevent cardiovascular events [28]. These values are within the range found in other works for marine fishes [15,24].

The hypocholesterolemic/hypercholesterolemic index (HH) is related with the effect of specific fatty acids on cholesterol metabolism, and higher values are considered more desirable for human health [29]. The highest HH value among all analyzed fish species was found for red cusk-eel (2.93) (Table 2), followed by Chilean hake (2.23), whereas the fishes with the lowest HH were Chilean sandperch (1.54) and jack mackerel (1.73). Recent works have reported HH values between 0.65 and 2.46 for fish lipids [15,24]. Considering both PUFA/SFA and HH indexes, the most favourable fish species from a nutritional point of view were red cusk-eel (1.60 and 2.93, respectively) and Chilean hake (1.52 and 2.23, respectively), whereas the less favourable was Chilean sandperch (0.80 and 1.54, respectively).

### 3.2. Lipid Amount and Fatty Acid Content in Shellfish

Lipid amount and fatty acid content in shellfish meat are shown in Table 3. Lipid content is generally lower than 2 g/100 g raw meat, except for sea squirt (2.96 g/100 g) and sea urchin (7.23 g/100 g). Lipid content in sea urchin is the highest among all analyzed species in this work for either fish or shellfish, and its value is similar to those reported in other sea urchin genders, such as 7.1% lipids content in fresh *Strongylocentrotus nudus* gonads from the Yellow Sea (China) [30].

Amounts of EPA + DHA ranged between 63.61 mg/100 g raw meat in Chilean abalone and 522.68 mg/100 g raw meat in sea squirt, which showed a notably higher amount than the other shellfish species analyzed. Other works have reported values of EPA + DHA in a wide range between 95 and 510 mg/100 g meat of several shellfishes, EPA being generally more abundant than DHA [31,32]. The same trend was observed in our study, and EPA was found to be more abundant than DHA in shellfishes, except in razor clam, where the amount of DHA was higher than that of EPA. The highest and lowest DHA/EPA ratios were found in razor clams (1.12) and sea urchins (0.05) respectively, which contained a very low amount of DHA. Actually, DHA amount found in sea urchins is the lowest among all marine species analyzed in this work (less than 10 mg/100 g meat), even considering its high lipid content. This fact is in agreement with previous evidence [33,34].

The sea squirt is the richest source of EPA + DHA among all assayed shellfish species, and according to our results, consumption of 47.8 g can provide 250 mg EPA + DHA (Table 3). It means that 3.3 servings per week (334.6 g) of sea squirt is able to supply the minimal recommended amount of EPA + DHA (1750 mg weekly). All other analyzed shellfishes require an unusually high consumption (more than 8 servings per week) to reach the recommended EPA + DHA intake, as their content of such n-3 PUFA is much lower.

Regarding SFA, palmitic acid is the most abundant in most shellfishes analyzed in this work, which is the usual trend in marine foods. However, in sea urchins, myristic acid (14:0) (1.76 g/100 g meat) is notably more abundant than 16:0 (1.02 g/100 g meat) (Table 3). The main food source of sea urchins is algae [35], and several studies about FA composition of algae in Chile and other parts of the world show that some of them contain a relevant amount of 14:0, being this FA and 16:0 much more abundant than DHA [36,37,38], which could explain the high amount of SFA and low amount of DHA found in sea urchin. Previous evidence also shows high levels of myristic and palmitic acids and low levels of DHA in sea urchins fed algae [38]. Because of the high contribution of SFA to total FA profile in sea urchins, the PUFA/SFA ratio in this species is 0.20, very low compared to other shellfishes, which are between 1.06 in clam and 2.10 in stone crab. Sea urchin is appreciated as seafood for the Chilean population, but due to its large supply of SFA, its consumption should be moderated. PUFA/SFA ratios for the rest of the analyzed shellfishes ranged between 1.06 for clam and 2.10 for stone crab, a similar range as those of analyzed fish species.

The n-6/n-3 ratio in shellfishes ranged between 0.04 (razor clam) and 0.17 (yellow squat lobster), except for sea urchin, which reached a value of 0.88 because of its high content of n-6 PUFA (280.3 mg/100 g raw meat) compared with the rest of analyzed shellfish species (Table 3). Such values were in the same range that those found in fish species (0.08–0.22). Regarding HH index, the lowest value among shellfishes was found in sea urchin (0.21) due to its large amount of SFA (Table 3). The other shellfish species showed HH values between 1.73 (sea squirt) and 4.75 (stone crab). Red and yellow squat lobster as well as stone crab had higher HH indexes than red cusk-eel, which was the fish species with the highest HH value (2.93).

### 3.3. Lipid Classes in Extracted Oils from Fish and Shellfish

**Figure 1:**
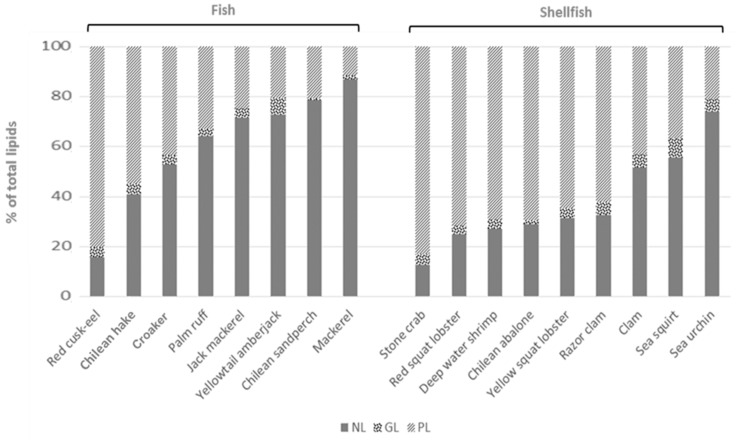
Proportions of lipid classes in total extracted lipids from fishes and shellfishes. NL: neutral lipids; GL: glycolipids; PL: phospholipids.

Extracted lipids from fish and shellfish were fractionated into NL, GL and PL, and results are shown in Figure 1. All lipids extracted from fishes were mainly composed (>50%) of NL except Chilean hake and red cusk-eel, which were mainly composed by PL (54.8 and 80.3% of total lipids, respectively). Mackerel and Chilean sandperch lipids were the richest in NL (86.9 and 78.4% of total lipids, respectively), followed by yellowtail amberjack and jack mackerel (72.7 and 71.4% NL of total lipids, respectively). Considering the amount of lipids contained in those fishes (Table 2), it is observed that the oilier the fish, the higher the amount of NL in their lipids. This can be explained taking into account that lipids accumulate in fish as triacylglycerols (TAG), which are the main class of NL usually found in animal tissues. The same trend is observed in shellfish, being sea squirt and sea urchin, the oilier species here analyzed, and also those with the highest NL content in their lipids (Figure 1, Table 3). Clam lipids were also mainly composed of NL (51.6% of total lipids). PL was found to be the main lipid class in the rest of the studied shellfish species. GL are a minor lipid component in both fishes and shellfishes, available in less than 5% of total lipids, except for clam, razor clam, yellowtail amberjack and sea squirt (5.1, 5.3, 6.8 and 7.7% of total lipids, respectively). In general, GL were found to be proportionally more abundant in shellfish than in fish. In shellfish, GL values ranged between 1.6 and 7.7% of total lipids in Chilean abalone and sea squirt, respectively, whereas in fish, values were between 0.8 and 6.8% of total lipids in Chilean sandperch and yellowtail amberjack, respectively. These numbers are in agreement with previous reports showing that the GL fraction in fish oil is lower than 10% of total lipids [39,40].

### 3.4. Fatty Acid Distribution among Lipid Classes

**Figure 2:**
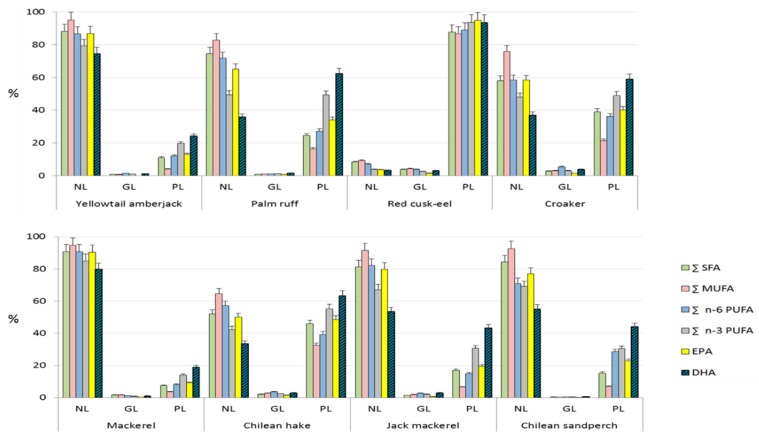
Distribution (% in each lipid class of total lipids) of SFA, MUFA, n-6 PUFA, n-3 PUFA, EPA and DHA among different lipid classes in extracted fish lipids. Σ n-3 PUFA includes EPA and DHA. Both n-3 PUFA are displayed separately because of their nutritional relevance. NL: neutral lipids; GL: glycolipids; PL: phospholipids.

**Figure 3:**
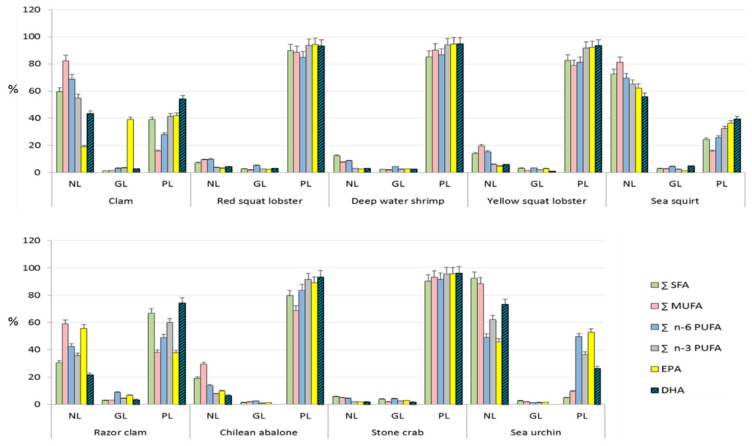
Distribution (% in each lipid class of total lipids) of SFA, MUFA, n-6 PUFA, n-3 PUFA, EPA and DHA among different lipid classes in extracted shellfish lipids. Σ n-3 PUFA includes EPA and DHA. Both n-3 PUFA are displayed separately because of their nutritional relevance. NL: neutral lipids; GL: glycolipids; PL: phospholipids.

In order to simplify the display of fatty acid profiles within lipid classes for each species, they were grouped into Σ SAT, Σ MUFA, Σ n-6 PUFA and Σ n-3 PUFA. EPA and DHA, though they were included within Σ n-3 PUFA, are displayed separately because of their particular relevance in the discussion. Results are shown in Figure 2 (fishes) and Figure 3 (shellfishes). These figures display the distribution (in %) of each FA or FA class among NL, GL and PL in each species.

DHA in fishes is mostly located in NL in four species: mackerel (79.8% of all DHA available in mackerel lipids are found in NL), yellowtail amberjack (74.6% DHA), Chilean sandperch (55.1% DHA) and jack mackerel (53.6% DHA) and in PL in the other four analyzed species: red cusk-eel (93.6% DHA), Chilean hake (67.2% DHA), palm ruff (62.4% DHA) and corvina drum (59.1% DHA). It is observed that DHA is significantly concentrated (*p*-value = 0.005) in NL in oily fishes (>2 g lipids/100 g raw fillet) and in PL (*p*-value < 0.001) in lean fishes (<2 g lipids/100 g raw fillet). In our previous work, the same fact was observed: DHA was mostly concentrated in PL in lean fishes, and only in Pacific sandperch, the species with the highest lipid content (2.3 g/100 g raw fillet), DHA was mainly concentrated in NL [7].

Regarding EPA, it is mainly found in the NL fraction in most analyzed fish species: mackerel (90.4% of all EPA available in mackerel lipids are found in NL), yellowtail amberjack (86.8% EPA), jack mackerel (79.9% EPA), Chilean sandperch (76.9% EPA), palm ruff (65.1% EPA), corvina drum (58.4% EPA). In Chilean hake, EPA was almost equally distributed between NL (50.0% EPA) and PL (48.5% EPA), and only in red cusk-eel EPA was found mainly in PL (94.9% EPA). It is observed that, for fishes with a lipid content higher than 1.3 g/100 g raw fillet, EPA is significantly mainly found in NL (*p*-value < 0.001), and only in red cusk-eel, with a very low lipid amount (0.69 g/100 g raw fillet), EPA is significantly concentrated in PL (*p*-value < 0.001).

In shellfish, DHA is mostly found as PL (Figure 3) (*p*-value < 0.001), except in sea squirt and sea urchin, the two shellfish species with the highest lipid content (Table 3), where DHA is mainly found as NL (*p*-value = 0.003). EPA is markedly found as PL in red and yellow squat lobster, deep water shrimp, Chilean abalone and stone crab (*p*-value < 0.001), and as NL in sea squirt and razor clam (*p*-value = 0.04). In sea urchin, EPA is almost equally distributed between NL and PL (*p*-value = 0.658). Interestingly, EPA was found as GL in a much larger proportion in clam than in the other analyzed species, both fishes and shellfishes. The reason for that is unclear, and unfortunately, we could not find any reference describing the distribution of fatty acids in this species. However, we speculate that the clams analyzed in this study may have assimilated EPA as GL through feeding microalgae with a high proportion of EPA-containing GL, as some microalgae species are rich in this type of lipid [41].

### 3.5. Nutritional Relevance for the Chilean Population

It is well established through the dietary guidelines that seafoods (including fish and shellfish) should be consumed at least twice a week because of their high content of n-3 PUFA EPA and DHA and their association with the prevention of non-communicable diseases. In Chile, however, this recommendation lacks detailed information regarding fatty acid profiles of commonly consumed seafood in the country, so decision-makers, health professionals and consumers do not know which marine resources can provide the highest content of these nutrients, and therefore cannot give consumers accurate recommendations of intake. This fact is critical because stroke and ischemic heart disease are leading causes of death among Chilean adults, and because specific groups of Chilean population, such as pregnant women, present a very low intake of n-3 PUFA [42]. This work provides a new insight that, together with our previous published data [7], seeks to contribute to improving the knowledge of the nutritional value of marine foods consumed in the country. We found that two servings per week of mackerel and yellowtail amberjack would be enough to provide the minimal required amount of EPA + DHA. Both species are fatty fishes where EPA and DHA are mainly available in the neutral lipid fraction. Shellfishes do not supply as much EPA and DHA as fishes, but in most of them, these n-3 PUFA are available as phospholipids, which may increase their bioaccessibility once they are ingested.

Fishes and shellfishes were obtained from local fishermen to reflect the way these products are sold in the Chilean market. Although this methodology limits the ability to monitor the quality of products from harvest to analysis, it was chosen because the target of the study was to analyze seafood once they are available for consumers.

## 4. Conclusions

This study expands the knowledge of fatty acid composition and distribution of Pacific seafood species commonly consumed in Chile. A very limited number of works have reported the fatty profiles of South Pacific marine fishes, but to our knowledge, this is the first study which shows the content and distribution of fatty acids in shellfish species captured in the South Pacific coast. This information provides a novel nutritional insight which is urgently needed to help nutritionists and other health professionals give more accurate counseling to the population to help them to reach the recommended daily intakes of n-3 PUFA, mainly EPA and DHA. This is a cross-sectional study, and seasonal variations regarding lipid content in marine foods due to biological, environmental, or feeding conditions must be considered in future works. Further research is recommended to estimate the content of other lipid components such as sterols and tocopherols.

## Figures and Tables

**Table 1:** Fish and shellfish species analyzed in this work.

	Common Name in English	Common Name in Chile	Scientific Name
**Fish**	Yellowtail amberjack	Palometa	*Seriola lalandi*
	Palm ruff	Cojinoba del Norte	*Seriolella violacea*
	Red cusk-eel	Congrio colorado	*Genypterus chilensis*
	Croaker	Corvina	*Cilus gilberti*
	Mackerel	Caballa	*Scomber japonicus*
	Chilean hake	Merluza común	*Merluccius gayi gayi*
	Jack mackerel	Jurel	*Trachurus murphyi*
	Chilean sandperch	Rollizo	*Pinguipes chilensis*
**Shellfish**	Clam	Almeja	*Venus antiqua*
	Red squat lobster	Langostino colorado	*Pleuroncodes monodon*
	Yellow squat lobster	Langostino amarillo	*Cervimunida johni*
	Deep water shrimp	Camarón nailon	*Heterocarpus reedi*
	Sea squirt	Piure	*Pyura chilensis*
	Razor clam	Macha	*Mesodesma donacium*
	Chilean abalone	Loco	*Concholepas concholepas*
	Stone crab	Jaiba marmola	*Cancer edwardsi*
	Sea urchin	Erizo	*Loxechinus albus*

**Table 2:** Total extracted lipids (g/100 g raw fillet) and amounts of each fatty acid (mg/100 g raw fillet) in fish. Data are reported as mean ± standard deviation. Nutritional indexes (HH, PUFA/SFA and n-6/n-3 ratios) and amounts of raw fish flesh needed to provide the minimal recommended daily amount of EPA + DHA (250 mg) for healthy adults are also reported.

	Yellowtail Amberjack	Palm Ruff	Red Cusk-eel	Croaker	Mackerel	Chilean Hake	Jack Mackerel	Chilean Sandperch
Total lipids (g/100 g fillet)	4.59 ± 0.30	1.71 ± 0.07	0.69 ± 0.06	1.44 ± 0.05	6.44 ± 0.21	1.29 ± 0.08	3.77 ± 0.22	2.72 ± 0.11
FA content (mg/100 g fillet)								
14:0	99.89 ± 3.63	52.42 ± 2.88	2.23 ± 0.02	37.16 ± 1.18	262.13 ± 20.52	24.96 ± 0.61	193.70 ± 6.41	93.14 ± 9.00
15:0	25.87 ± 1.00	13.90 ± 0.71	0.86 ± 0.06	5.21 ± 0.25	62.67 ± 5.37	3.77 ± 0.09	20.58 ± 0.64	14.49 ± 1.51
16:0	794.74 ± 22.98	262.49 ± 11.19	55.60 ± 2.99	208.88 ± 7.02	1248.20 ± 75.94	166.52 ± 3.65	688.38 ± 17.62	593.53 ± 54.78
17:0	29.51 ± 1.07	10.39 ± 0.41	1.17 ± 0.10	4.95 ± 0.25	53.24 ± 3.22	3.60 ± 0.11	13.55 ± 1.25	11.48 ± 1.15
18:0	295.55 ± 8.64	117.31 ± 4.77	25.81 ± 1.43	65.85 ± 2.41	416.72 ± 22.62	41.94 ± 0.57	201.07 ± 3.74	138.33 ± 11.11
20:0	9.72 ± 0.38	5.18 ± 0.18	0.36 ± 0.01	2.53 ± 0.09	19.57 ± 1.35	1.77 ± 0.07	6.80 ± 0.60	6.65 ± 1.08
24:0	4.52 ± 0.21	0.00 ± 0.00	0.34 ± 0.04	1.41 ± 0.11	3.00 ± 0.38	1.34 ± 0.18	2.06 ± 0.27	1.94 ± 0.17
Σ SFA	1259.78 ± 24.87	461.69 ± 12.53	86.36 ± 3.32	326.00 ± 7.53	2065.53 ± 82.10	243.91 ± 3.76	1126.14 ± 19.18	859.56 ± 56.56
16:1n-7	165.81 ± 7.83	35.97 ± 1.07	5.61 ± 0.24	61.76 ± 3.29	198.98 ± 17.29	33.68 ± 0.76	169.38 ± 5.68	319.91 ± 35.98
18:1n-9	803.83 ± 23.33	235.10 ± 10.49	32.67 ± 1.66	97.50 ± 4.61	1255.30 ± 78.65	70.94 ± 1.44	533.70 ± 15.89	382.75 ± 38.68
18:1n-7	102.16 ± 3.07	47.09 ± 2.44	9.68 ± 0.53	34.69 ± 1.46	257.38 ± 16.41	34.66 ± 1.04	115.04 ± 2.57	127.39 ± 12.64
20:1n-9	41.84 ± 1.12	27.90 ± 1.34	1.17 ± 0.03	8.86 ± 0.47	155.43 ± 9.58	5.94 ± 0.43	60.77 ± 2.57	25.27 ± 2.50
24:1n-9	33.41 ± 0.80	12.30 ± 0.50	1.78 ± 0.08	5.96 ± 0.08	30.41 ± 1.49	6.58 ± 0.94	20.75 ± 0.64	8.20 ± 0.91
Σ MUFA	1147.04± 24.84	358.35 ± 10.92	50.92 ± 1.76	208.77 ± 5.87	1897.50 ± 82.75	151.79 ± 2.19	899.65 ± 17.27	863.50 ± 54.39
18:2n-6	55.89 ± 6.52	8.87 ± 0.45	2.76 ± 0.11	15.94 ± 0.99	129.64 ± 8.13	17.94 ± 0.80	71.03 ± 1.90	32.11 ± 3.24
20:4n-6	31.98 ± 3.06	70.63 ± 2.79	10.95 ± 0.99	15.52 ± 0.46	99.85 ± 4.69	11.66 ± 0.28	29.26 ± 0.83	70.02 ± 4.94
18:3n-3	22.41 ± 4.00	4.13 ± 0.16	0.36 ± 0.05	3.19 ± 0.29	42.79 ± 3.41	5.52 ± 0.14	15.47 ± 0.44	5.91 ± 0.56
18:4n-3	35.25 ± 5.87	9.02 ± 0.51	0.37 ± 0.03	10.45 ± 0.67	97.78 ± 8.48	10.51 ± 0.31	48.75 ± 1.77	8.59 ± 0.90
20:4n-3	22.24 ± 0.78	6.69 ± 0.43	0.61 ± 0.08	5.82 ± 0.30	37.66 ± 3.04	3.89 ± 0.10	23.97 ± 0.57	8.07 ± 0.58
20:5n-3 (EPA)	154.41 ± 6.53	84.01 ± 5.11	40.76 ± 2.01	106.78 ± 5.10	414.72 ± 30.21	121.38 ± 5.92	282.01 ± 8.58	285.87 ± 22.97
22:5n-3	80.63 ± 3.10	37.25 ± 2.16	7.61 ± 0.52	30.65 ± 1.28	128.06 ± 7.67	10.92 ± 0.43	92.57 ± 1.80	58.00 ± 3.96
22:6n-3 (DHA)	761.35 ± 18.56	220.02 ± 13.20	74.39 ± 5.82	187.80 ± 7.13	955.95 ± 46.90	188.00 ± 3.37	504.88 ± 7.57	221.73 ± 10.66
Σ PUFA	1164.17 ± 22.36	440.63 ± 14.61	137.81 ± 6.26	376.15 ± 8.96	1906.45 ± 57.90	369.81 ± 6.89	1067.96 ± 11.92	690.30 ± 26.33
Σ n-6 PUFA	87.87 ± 7.20	79.50 ± 2.83	13.71 ± 0.99	31.46 ± 1.09	229.49 ± 9.39	29.60 ± 0.84	100.30 ± 2.07	102.13 ± 5.90
Σ n-3 PUFA	1076.29 ± 21.17	361.13 ± 14.33	124.10 ± 6.18	344.69 ± 8.89	1676.95 ± 57.13	340.21 ± 6.83	967.66 ± 11.74	588.17 ± 25.66
Σ EPA + DHA	915.76 ± 19.68	304.04 ± 14.15	115.15 ± 6.16	294.57 ± 8.76	1370.67 ± 55.79	309.38 ± 6.81	786.90 ± 11.44	507.60 ± 25.32
PUFA/SFA	0.92	0.95	1.60	1.15	0.92	1.52	0.95	0.80
n-6/n-3 ratio	0.08	0.22	0.11	0.09	0.14	0.09	0.10	0.17
HH	2.14	2.10	2.93	1.86	2.00	2.23	1.73	1.54
Amount of flesh (g) to supply 250 mg EPA + DHA	27.3	82.2	217.1	84.9	18.2	80.8	31.8	49.3

**Table 3:** Total extracted lipids (g/100 g raw meat) and amounts of each fatty acid (mg/100 g raw meat) in shellfish. Data are reported as mean ± standard deviation. Nutritional indexes (HH, PUFA/SFA and n-6/n-3 ratios) and amounts of raw fish flesh needed to provide the minimum recommended daily amount of EPA + DHA (250 mg) for healthy adults are also reported.

	Clam	Red Squat Lobster	Yellow Squat Lobster	Deep Water Shrimp	Sea Squirt	Razor Clam	Chilean Abalone	Stone Crab	Sea Urchin
Total lipids (g/100 g meat)	1.47 ± 0.12	1.23 ± 0.08	1.32 ± 0.07	1.35 ± 0.06	2.96 ± 0.25	1.94 ± 0.07	0.87 ± 0.04	1.20 ± 0.16	7.23 ± 0.41
FA content (mg/100 g meat)									
14:0	19.70 ± 0.65	3.66 ± 0.17	2.86 ± 0.16	7.75 ± 0.23	96.76 ± 7.13	20.63 ± 1.23	5.94 ± 0.30	2.02 ± 0.27	1762.43 ± 74.47
15:0	5.60 ± 0.24	12.09 ± 0.18	2.42 ± 0.02	5.58 ± 0.25	23.98 ± 3.25	3.97 ± 0.34	0.29 ± 0.03	1.45 ± 0.07	22.12 ± 1.83
16:0	130.30 ± 5.89	73.52 ± 2.01	72.34 ± 1.21	93.06 ± 2.17	314.73 ± 19.17	113.71 ± 7.57	43.60 ± 3.69	62.19 ± 2.21	1022.63 ± 51.13
17:0	50.57 ± 2.06	9.69 ± 0.26	4.67 ± 0.03	5.05 ± 0.26	12.66 ± 0.80	2.79 ± 0.21	25.63 ± 2.17	8.68 ± 0.33	35.49 ± 1.45
18:0	56.03 ± 3.03	28.90 ± 0.74	23.50 ± 0.06	29.67 ± 0.53	77.97 ± 4.03	62.99 ± 4.74	25.31 ± 1.07	39.06 ± 1.92	91.87 ± 4.72
20:0	1.11 ± 0.03	2.96 ± 0.17	4.66 ± 0.17	1.85 ± 0.14	7.85 ± 0.20	1.64 ± 0.08	0.59 ± 0.03	1.01 ± 0.10	27.94 ± 1.54
24:0	4.41 ± 0.31	0.79 ± 0.09	0.50 ± 0.08	1.04 ± 0.11	0.00 ± 0.00	0.67 ± 0.06	0.25 ± 0.02	0.00 ± 0.00	0.00 ± 0.00
Σ SFA	267.73 ± 6.98	131.61 ± 2.18	110.96 ± 1.23	144.00 ± 2.28	533.94 ± 21.22	206.40 ± 9.02	101.62 ± 4.43	114.40 ± 2.96	2962.48 ± 90.51
16:1n-7	52.13 ± 2.14	4.90 ± 0.14	0.79 ± 0.04	3.57 ± 0.07	152.64 ± 11.13	26.16 ± 2.11	3.76 ± 0.27	21.81 ± 1.07	138.93 ± 6.46
18:1n-9	17.47 ± 0.86	65.51 ± 2.04	62.92 ± 0.96	82.66 ± 1.22	81.74 ± 3.02	24.41 ± 1.24	8.69 ± 0.50	66.37 ± 2.37	71.71 ± 2.79
18:1n-7	24.35 ± 1.32	20.43 ± 0.66	21.60 ± 0.34	33.46 ± 0.98	147.60 ± 9.35	19.28 ± 1.53	5.56 ± 0.58	24.91 ± 1.10	571.99 ± 30.34
20:1n-9	11.75 ± 0.57	2.20 ± 0.22	1.48 ± 0.07	0.50 ± 0.04	9.40 ± 0.62	14.49 ± 0.97	7.46 ± 0.34	1.62 ± 0.06	444.43 ± 33.93
24:1n-9	0.00 ± 0.00	0.86 ± 0.14	0.44 ± 0.02	1.77 ± 0.16	0.00 ± 0.00	0.00 ± 0.00	0.00 ± 0.00	0.00 ± 0.00	2.89 ± 0.30
Σ MUFA	105.70 ± 2.72	93.91 ± 2.17	87.23 ± 1.02	121.96 ± 1.57	391.38 ± 15.01	84.35 ± 3.05	25.47 ± 0.88	114.71 ± 2.83	1229.95 ± 46.05
18:2n-6	2.57 ± 0.06	5.02 ± 0.20	4.55 ± 0.26	5.42 ± 0.07	51.11 ± 2.23	1.66 ± 0.03	7.31 ± 0.93	7.60 ± 0.41	10.60 ± 0.39
20:4n-6	19.82 ± 0.66	20.19 ± 0.54	24.93 ± 0.23	13.33 ± 0.38	18.25 ± 1.50	10.03 ± 0.71	6.50 ± 0.30	15.12 ± 0.93	269.72 ± 13.32
18:3n-3	5.81 ± 0.80	0.17 ± 0.04	1.42 ± 0.05	1.93 ± 0.07	25.60 ± 1.80	1.71 ± 0.20	0.24 ± 0.01	0.76 ± 0.04	5.15 ± 0.57
18:4n-3	10.32 ± 0.42	1.97 ± 0.06	1.64 ± 0.04	0.91 ± 0.09	71.44 ± 5.43	7.25 ± 0.57	1.60 ± 0.15	0.66 ± 0.03	69.15 ± 2.88
20:4n-3	6.08 ± 0.41	0.85 ± 0.15	0.88 ± 0.07	0.94 ± 0.09	0.00 ± 0.00	4.54 ± 0.40	0.48 ± 0.01	1.23 ± 0.03	27.08 ± 1.34
20:5n-3 (EPA)	130.44 ± 5.74	106.31 ± 3.00	90.58 ± 1.23	119.53 ± 3.43	356.97 ± 24.71	102.12 ± 7.55	46.70 ± 0.12	140.85 ± 5.80	199.01 ± 10.19
22:5n-3	25.13 ± 1.33	3.55 ± 0.14	4.88 ± 0.24	2.77 ± 0.08	12.29 ± 0.87	33.86 ± 1.55	38.31 ± 0.41	9.70 ± 0.56	8.40 ± 0.53
22:6n-3 (DHA)	83.91 ± 4.87	83.53 ± 2.23	72.33 ± 2.54	67.45 ± 1.80	165.71 ± 13.21	114.84 ± 6.18	16.91 ± 0.40	64.77 ± 2.16	9.54 ± 1.40
Σ PUFA	283.27 ± 7.73	221.59 ± 3.79	201.22 ± 2.86	212.28 ± 3.90	701.37 ± 28.74	276.01 ± 9.93	118.06 ± 1.15	240.69 ± 6.30	598.66 ± 17.15
Σ n-6 PUFA	22.39 ± 0.66	25.21 ± 0.58	29.48 ± 0.35	18.75 ± 0.39	69.36 ± 2.69	11.69 ± 0.72	13.81 ± 0.97	22.72 ± 1.02	280.32 ± 13.33
Σ n-3 PUFA	260.89 ± 7.70	196.38 ± 3.74	171.73 ± 2.84	193.53 ± 3.88	632.00 ± 28.61	264.32 ± 9.91	104.25 ± 0.61	217.97 ± 6.22	318.34 ± 10.79
Σ EPA + DHA	214.34 ± 7.52	189.83 ± 3.74	162.90 ± 2.83	186.98 ± 3.88	522.68 ± 28.02	216.96 ± 9.76	63.61 ± 0.42	205.62 ± 6.19	208.55 ± 10.28
PUFA/SFA	1.06	1.68	1.81	1.47	1.31	1.34	1.16	2.10	0.20
n-6/n-3 ratio	0.09	0.13	0.17	0.10	0.11	0.04	0.13	0.10	0.88
HH	1.90	3.68	3.48	2.91	1.73	2.15	2.52	4.75	0.21
Amount of meat (g) to supply 250 mg EPA + DHA	116.6	131.7	153.5	133.7	47.8	115.2	393.0	121.6	119.9
